# Insomnia Among Healthcare Workers Following SARS-CoV-2 Infection

**DOI:** 10.1192/j.eurpsy.2025.2326

**Published:** 2025-08-26

**Authors:** E. Bechrifa, D. Brahim, I. Youssef, N. Mechergui, H. Ben Said, M. Mersni, G. Bahri, M. Bani, N. Laadhari

**Affiliations:** 1 Occupational Medicine Department, Charles Nicolle Hospital; 2 Occupational Medicine Department, Mongi Slim Hospital; 3 Occupational Medicine Department, Habib Thameur Hospital; 4 Occupational Medicine Department, Maternity and Neonatology Center, Tunis, Tunisia

## Abstract

**Introduction:**

COVID-19 infections had a variety of symptoms, including a range of sleep disorders such as insomnia.

**Objectives:**

The objective of the study was to assess the prevalence of insomnia among healthcare workers (HCWs) following COVID-19 infection and to identify factors associated with its persistence over time.

**Methods:**

This prospective descriptive study was conducted at Charles-Nicolle Hospital in Tunis, monitoring HCWs infected with COVID-19 over a six-month period (January-July 2022). Insomnia severity was evaluated at infection onset (T0), at three months, and at six months post-infection, using the Insomnia Severity Index (ISI) in its French version. The presence of anxiety and depression was assessed via the Hospital Anxiety and Depression Scale (HAD) to determine potential associations.

**Results:**

The study included 155 healthcare workers, with an average age of 40.2 ± 10.3 years and an average work tenure of 14,1 ± 10 years. At baseline (J0), 42.5% of participants reported no insomnia, 21.3% had mild sub-clinical insomnia, 31% had moderate clinical insomnia, and 5.2% experienced severe clinical insomnia. At three months, the rates shifted, with 11.6% reporting no insomnia, 35.5% experiencing mild sub-clinical insomnia, and 7.1% having moderate clinical insomnia. By six months, 7.7% had no insomnia, 6.5% reported mild sub-clinical insomnia, and 3.9% continued to experience moderate clinical insomnia. Factors significantly associated with insomnia included a history of discopathy (p < 10⁻³), hospitalization (p < 10⁻³), and the death of a close relative due to COVID-19 (p = 0.012). Additionally, significant associations were found between persistent insomnia and anxiety and/or depression (p = 0.03).

**Conclusions:**

Persistent insomnia post-COVID-19 infection in HCWs underscores the importance of integrating mental health and sleep quality interventions into healthcare protocols to improve overall well-being and aid in their professional recovery.

**Disclosure of Interest:**

None Declared

